# Early biomarkers of extracapsular extension of prostate cancer using MRI-derived semantic features

**DOI:** 10.1186/s40644-022-00509-8

**Published:** 2022-12-23

**Authors:** Adalgisa Guerra, Filipe Caseiro Alves, Kris Maes, Steven Joniau, João Cassis, Rui Maio, Marília Cravo, Helena Mouriño

**Affiliations:** 1 Radiology Department, Hospital da Luz Lisboa, Avenida Lusíada, n° 100, 1500-650 Lisbon, Portugal; 2 Faculty of Medicine, Clinical Research CIBIT/ICNAS, University of Coimbra, 3000-548 Coimbra, Portugal; 3 Urology Department, Hospital da Luz Lisboa, Avenida Lusíada, n° 100, 1500-650 Lisbon, Portugal; 4 Urology Department, University Hospitals Leuven, UZ Leuven gasthuisberg campus, Urology, Herestraat 49, 3000 Leuven, Belgium; 5 Pathology Department, Hospital da Luz Lisboa, Avenida Lusíada, n° 100, 1500-650 Lisbon, Portugal; 6 Nova Medical School-Nova University of Lisbon, Portugal e Hospital da Luz Lisboa, Campo Mártires da Pátria, n° 130, 1169-056 Lisbon, Portugal; 7 Gastroenterology Department- Hospital da Luz Lisboa, Avenida Lusíada, n° 100, 1500-650 Lisbon, Portugal; 8 Centro de Estatística e Aplicações, Departamento de Estatística e Investigação Operacional, Faculdade de Ciências, Universidade de Lisboa, Edifício C6 – Piso 4, Campo Grande, 1749 – 016 Lisbon, Portugal

**Keywords:** Extracapsular extension, Prostate cancer, Magnetic resonance imaging, Radical prostatectomy, Staging, Capsular contact, Sematic features

## Abstract

**Background:**

To construct a model based on magnetic resonance imaging (MRI) features and histological and clinical variables for the prediction of pathology-detected extracapsular extension (pECE) in patients with prostate cancer (PCa).

**Methods:**

We performed a prospective 3 T MRI study comparing the clinical and MRI data on pECE obtained from patients treated using robotic-assisted radical prostatectomy (RARP) at our institution. The covariates under consideration were prostate-specific antigen (PSA) levels, the patient’s age, prostate volume, and MRI interpretative features for predicting pECE based on the Prostate Imaging–Reporting and Data System (PI-RADS) version 2.0 (v2), as well as tumor capsular contact length (TCCL), length of the index lesion, and prostate biopsy Gleason score (GS). Univariable and multivariable logistic regression models were applied to explore the statistical associations and construct the model. We also recruited an additional set of participants—which included 59 patients from external institutions—to validate the model.

**Results:**

The study participants included 184 patients who had undergone RARP at our institution, 26% of whom were pECE+ (i.e., pECE positive). Significant predictors of pECE+ were TCCL, capsular disruption, measurable ECE on MRI, and a GS of ≥7(4 + 3) on a prostate biopsy. The strongest predictor of pECE+ is measurable ECE on MRI, and in its absence, a combination of TCCL and prostate biopsy GS was significantly effective for detecting the patient’s risk of being pECE+. Our predictive model showed a satisfactory performance at distinguishing between patients with pECE+ and patients with pECE−, with an area under the ROC curve (AUC) of 0.90 (86.0–95.8%), high sensitivity (86%), and moderate specificity (70%).

**Conclusions:**

Our predictive model, based on consistent MRI features (i.e., measurable ECE and TCCL) and a prostate biopsy GS, has satisfactory performance and sufficiently high sensitivity for predicting pECE+. Hence, the model could be a valuable tool for surgeons planning preoperative nerve sparing, as it would reduce positive surgical margins.

## Background

Prostate cancer (PCa) is one of the most common cancers in Portugal and the world, with approximately 6600 new cases diagnosed per year in Portugal alone [1]. Despite the increasing incidence of PCa, a decline in global mortality has been observed in most regions of the world. Screening and improvements in PCa treatment may contribute to the declining trend in overall mortality, which is unrelated to the number of cases diagnosed [2].

The staging of PCa is the most critical factor in the prognosis of the disease and plays a decisive role in the selection of the most appropriate therapeutic approach to be taken in each clinical case. Radical prostatectomy (RP) is a well-established treatment for the management of localized PCa. The goal of RP is to achieve negative surgical margins while preserving urinary continence and erectile function. Therefore, accurate preoperative staging is crucial for guiding treatment [3]. The presence of extracapsular extension (ECE) is associated with adverse clinicopathological features and a significantly elevated risk of systemic progression and mortality following RP [4]. The presence of ECE can also lead to the recommendation of adjuvant radiotherapy (RT) after RP and thus can influence treatment decision, relevantly. The presence of ECE has been evaluated using only digital rectal examination and prostate-specific antigen (PSA) levels [3].

Multiparametric magnetic resonance imaging (mpMRI), which combines conventional anatomical and functional sequences, has been widely utilized in the detection and local staging of PCa, and treatment planning for patients with PCa [5–7]. Systematic reviews and meta-analyses have been performed regarding the different levels of mpMRI accuracy for ECE detection [8, 9]. In their recent meta-analysis, [10] analyzed the 42 quality-matched studies available in the literature and concluded that MRI scans lacked sensitivity (57%) to detect ECE, particularly when the scan results were read by radiologists with limited experience [10].

In the literature, several mpMRI features have been described as being associated with pathology-detected extracapsular extension (pECE). These features include curvilinear contact length, capsular irregularity, capsular bulging, obliteration of the rectoprostatic angle, asymmetry of the neurovascular bundles, invasion of periprostatic fat, MRI measurable ECE, and seminal vesicle invasion. The European Society of Urogenital Radiology (ESUR) score, published by the ESUR Prostate Working Group, uses a five-point grading scale based on MRI features to predict the presence of pECE in patients with PCa [11]. However, validation studies for these imaging markers are still sparse [6, 12, 13]. The ESUR score consists only of qualitative descriptors for predicting ECE and shows moderate intra-reader and inter-reader agreement (*k* = 0.61 and *k* = 0.63, respectively) in external validation studies [14]. Therefore, it is essential to research and validate reproducible unsubjective imaging features to improve mpMRI accuracy. These would reduce inter-reader variability and eliminate the need for laborious and subjective interpretation.

The purpose of this study is to evaluate the MRI morphostructural (semantic) features used in the detection of ECE and construct a predictive model that incorporates clinical findings, prostate biopsy results, and a proposed set of objective MRI variables to predict ECE in patients with prostate cancer (PCa) before RP.

## Materials and methods

This prospective single-center study compares clinical, mpMRI, and presurgical prostate biopsy parameters with final surgical pathology findings in patients with PCa who were treated using robotic assisted radical prostatectomy (RARP) at Hospital da Luz Lisbon.

**Fig. 1:**
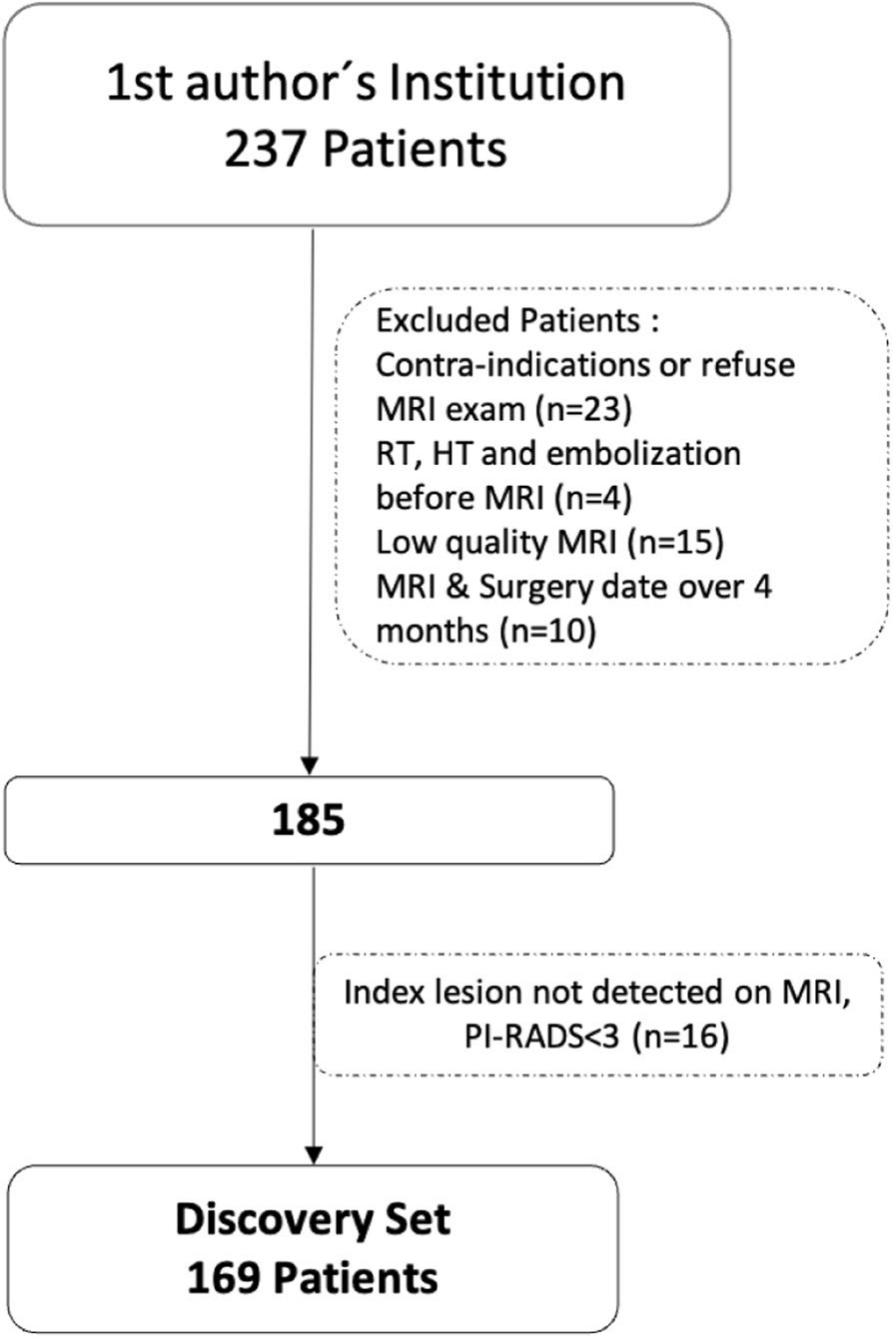
Flowchart of the patient selection process

This study included 257 participants, all of whom were patients diagnosed with PCa between 2015 and 2018. Each participant had a presurgical prostate biopsy Gleason score (GS) of ≥6 and underwent RARP at Hospital da Luz Lisbon. We performed MRIs using a standardized protocol on a 3 T MRI scanner. The exclusion criteria (Fig. 1) led to 72 patients being excluded, leaving only 185 patients for analysis. Sixteen patients from the group of 185 were excluded from the final statistical analysis because there was no index lesion detected in their MRI scans, indicated by a Prostate Imaging Reporting and Data System (PI-RADS) score of < 3, which precluded the use of MRI features. Therefore, a final cohort of 169 patients was used to build the predictive model. The predictive model was then evaluated on a validation group of 59 patients selected from seven external institutions using either 1.5 T or 3 T MRI scanners. The validation cohort also underwent RARP at Hospital da Luz between 2018 and 2020 under the same conditions as the original test group.

### MR imaging technique

All patients in the modeling cohort underwent mpMRI on a 3 T MRI device (Magnetom Verio and Magnetom Vida; Siemens Healthcare, Erlangen, Germany) using a pelvic phased-array coil. All MRI examinations included multiplanar T2-weighted turbo spin echo (TSE) imaging, axial T1-weighted TSE imaging, and axial diffusion-weighted imaging (DWI) of the prostate, with the DWI performed using *b*-values of 50,1000 sec/mm^2^ and 1500 to 2000 sec/mm^2^, with inline reconstruction of the apparent diffusion coefficient (ADC) map. Dynamic contrast-enhanced MRI (DCE-MRI) of the prostate was performed following administration of 0.1 mmol/kg gadopentetate dimeglumine (Magnevist; Bayer HealthCare Pharmaceuticals, Montville, NJ). The contrast agent was administered as an intravenous bolus using a power injector, followed by a 20 mL saline flush, both administered at a 3 mL/sec injection rate. A biexponential semiquantitative model was applied to the DCE-MRI acquisition to generate two parametric maps representing the mathematically derived maximum slope of enhancement during contrast-enhanced acquisition (maximum slope map) and washout following peak enhancement (washout map). The patients from the external validation cohort were examined using a standardized mpMRI protocol that included T2-weighted turbo or fast spin echo sequences in all three orthogonal planes and a DWI sequence with at least 2 *b*-values (the highest *b*-value was equal to or higher than 1000 s/mm^2^ and the lowest *b*-values were between 0 and 50 s/mm^2^. All patients in the original test group and the external validation group were operated at Hospital da Luz Lisbon by the same surgical team (KM), and the prostate specimens were analyzed by the same pathologist. The covariates used in the statical model were presurgical MRI semantic features, clinical variables, and biopsy-derived variables. The dependent variable was the presence of pECE.

### MRI analysis

**Fig. 2:**
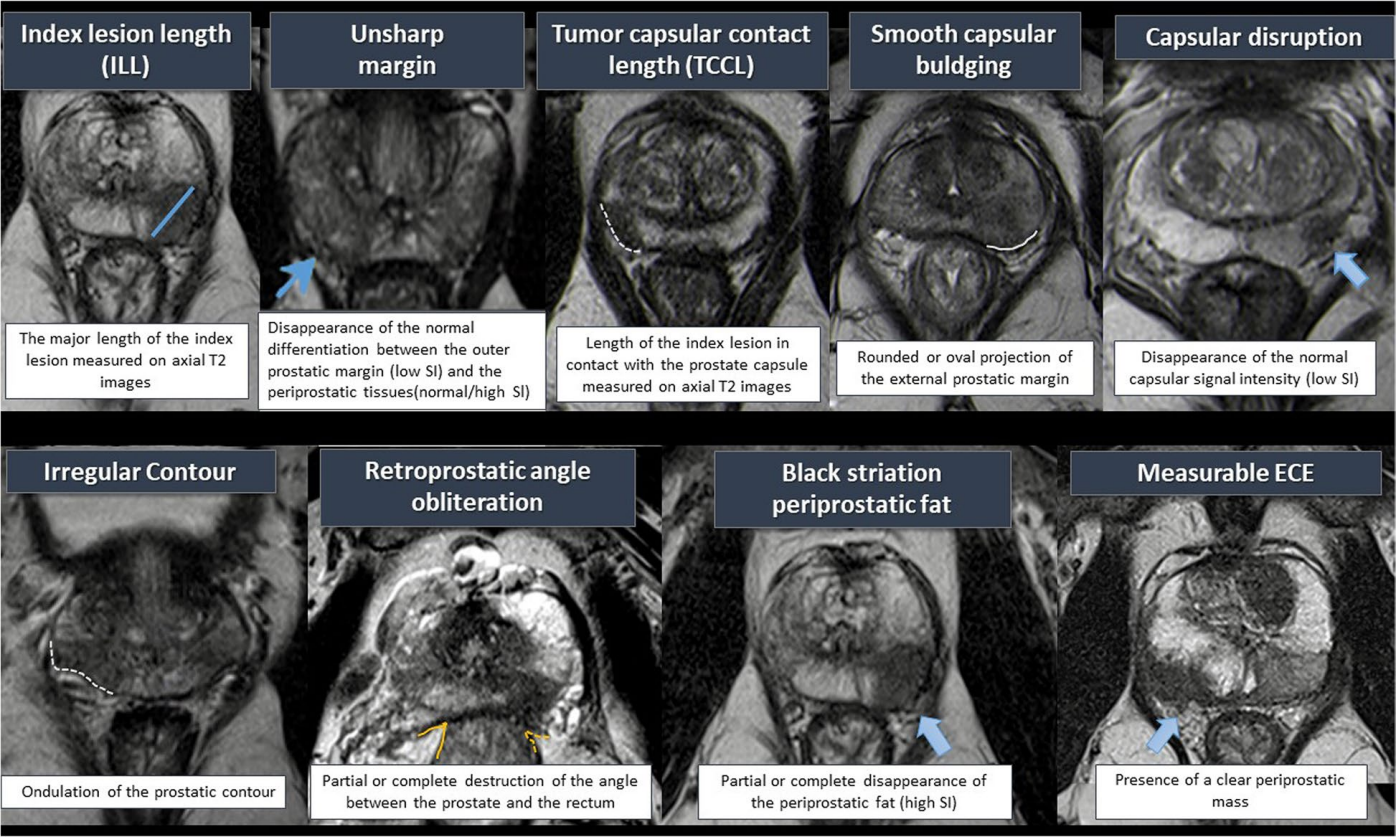
MRI Semantic features for detection of pECE

We analyzed the MRI scan results, focusing only on index lesions defined as the largest nodule for multifocal disease and/or the most aggressive tumor (the highest PI-RADS in contact with the capsule), which would dictate the broad clinical behavior of the PCa and contact with the prostate capsule. The index lesion was identified and classified based on the PI-RADS version 2.0 (v2) score [6]. We also analyzed the interpretative features set (black striation periprostatic fat, obliteration of the rectoprostatic angle, measurable ECE on MRI, smooth capsular bulging, capsular disruption, unsharp margin, and irregular contour) used for the identification of pECE on MRI (Fig. 2).

In addition, we included two proposed new variables to the MRI features, which can be easily quantified: the index lesion length (ILL), which corresponds to the major length of the index lesion; and the tumor capsular contact length (TCCL), which is the contact length of the index lesion with the prostate capsule. Both were measured in millimeters on axial T2 images (Fig. 2), and we used a curvilinear ruler to draw the TCCL.

All examinations were evaluated by an investigator (AG) with 10 years of urological radiology experience, using the commercial syngo.via software (Siemens Healthineers) and the standardized protocol adopted at our institution, allowing synchronization and simultaneous display on two monitors. The sequence primarily used to assess all semantic features was T2-weighted imaging (WI). DWI and ADC maps were used to detect and classify the index lesion. DCE-MRI was used only for lesion classification based on PI-RADS v2. To perform a reproducibility analysis, all semantic features were also evaluated by a second radiologist with one year of experience in MRI. For the reproducibility analysis, the interclass correlation coefficient (ICC) was computed, and all semantic features below a threshold of 0.75 were considered unreproducible. The two radiologists were blinded to all information on the pathology findings and the initial mpMRI results.

### Clinical and histological analysis

All prostate biopsies performed outside our institution were reviewed in accordance with our presurgical protocol.

Histology sections from prostatectomy specimens and the prostate biopsy were processed in accordance with the International Society of Urological Pathology (ISUP) Consensus Guidelines.

The prostatectomy specimens were sliced from apex to base at 4–6 mm intervals in a plane perpendicular to the prostatic urethra. The distal portions of the apex and the proximal parts of the base were amputated at 4 mm intervals and sliced longitudinally to assess the caudal and cranial surgical margins. After fixation in 5% buffered formalin, each block was processed and embedded in paraffin, and then 4–5 μm sections were cut with a microtome and stained with hematoxylin–eosin.

Tumors were classified as pECE negative (pECE−) if no ECE was detected, and pECE positive (pECE+) if pECE was detected. A pECE+ diagnosis implied the presence of a tumoral extension beyond the periphery of the prostate gland.

Comparisons for visual correlations between the histology and MRI images of the index lesion were performed by a pathologist (JC) and a radiologist (AG) to determine the presence of a tumor. We excluded 16 cases in which the index lesion was not apparent on MRI (PI-RADS < 3). Correlated cases were considered if the index lesion detected via MRI was also detected on pathology and defined as the largest lesion. Cases were also considered as correlated when the index lesion observed via MRI was also detected on pathology, despite multifocality.

The clinical and laboratory data evaluated included the age of the patients, PSA levels, PSA density (PSA/prostate volume), and mpMRI and surgery dates. Data on the patients was anonymized and then collated in an Excel database and organized according to the surgery dates.

### Statistical analysis

The binary outcome variable in this study is the presence or absence (i.e., 1 or 0) of pECE on the pathological prostate specimen after a prostatectomy (i.e., pECE+ or pECE−). The covariates under consideration, as continuous covariables, were age at MRI (in years), prostate volume (grams), PSA levels (ng/ml), PSA density (ng/ml/g), PSA divided by the volume of the prostate gland, ILL (mm), and capsular contact length (mm). The PI-RADS v2 (1–5, prostate biopsy GS, smooth capsular bulging, irregular contour, capsular disruption, unsharp margin, black striation periprostatic fat, obliteration of the rectoprostatic angle, and measurable ECE were all binary variables, such that 1 = present and 0 = absent (Fig. 2). The GS was categorized into two groups for statistical analysis: the GS of marginally aggressive tumors were 6 (3 + 3) and 7 (3 + 4), while the GS of fully aggressive tumors were 7 (4 + 3), 8 (4 + 4), and 9 (4 + 5 or 5 + 4). The index lesions analyzed were classified according to PI-RADS v2 scores 3, 4, and 5. The diagnostic performances of the covariables used to discriminate pECE+ patients from pECE− patients were evaluated using odds ratios (Ors) in both univariable and multivariable logistic regression analyses. A *p*-value of < 0.05 was considered statistically significant. The receiver operating characteristic (ROC) curve was computed to evaluate the overall performance of the predictive model in terms of sensitivity and specificity, and the area under the ROC curve (AUC) was assessed. The cutoff point considered in these computations was obtained from the Youden index, i.e., the cutoff point is the value that maximizes the differentiating ability of the model when equal weight is given to sensitivity and specificity [15–17]. Confidence intervals for these measures were computed, and model fit was also assessed using the Hosmer and Lemeshow test [17]. Furthermore, we applied the final model to the validation group and estimated the pECE probabilities to validate our predictive model. The main results from the validation group were compared to those in the original dataset, Ih allowed us to determine the goodness-of-fit of the final model. The data analysis was performed using the R package (version 4.0.4).

## Results

Based on the surgical specimens from the 169 patients, 44 patients (26%) were pECE+ and 125 patients (74%) were pECE−. The following are summarized in Table 1: the main clinical group characteristics, the imaging semantic features, and the proposed MRI variables in both pECE+ patients and pECE− patients.

**Table 1:** Characteristics of patients by the presence of pECE in prostatectomy specimen (sample size = 169)

Variables	pECE+	pECE-	*p-value*
	(n.° of patients = 44)	(n.° of patients = 125)	
Group characteristics			
Age at MRI (years)	60.7 ± 7.2 (45.7; 72.4)	61.9 ± 6.7 (41.2; 75.8)	0.334
Prostate volume (g)	38.8 ± 21.2 (20; 148)	42.8 ± 19.7 (18; 122)	0.282
PSA (ng/ml)	8.0 ± 4.6 (2.2; 21.2)	7.0 ± 3.9 (2.2; 31.0)	0.212
PSAD* (ng/ml/g)	0.2 ± 0.2 (0.1; 1.0)	0.2 ± 0.1 (0.0; 0.8)	0.090
Index lesion PI-RADS V2			
3	1 (2.27)	9 (7.20)	
4	14 (31.82)	77 (61.60)	0.000
5	29 (65.91)	39 (31.20)	
Gleason score in prostate biopsy			
Less Aggressive <= 7 (3 + 4)	14 (31.82)	106 (84.80)	0.000
More Aggressive > = 7 (4 + 3)	30 (68.18)	19 (15.20)	
Semantic features on MRI			
Smooth capsular bulging			
No	5 (11.36)	61 (48.80)	0.000
Yes	39 (88.64)	64 (51.20)	
Capsular disruption			
No	6 (13.64)	81 (64.80)	0.000
Yes	38 (86.36)	44 (35.20)	
Unsharp margin			
No	8 (18.18)	72 (57.60)	0.000
Yes	36 (81.82)	53 (42.40)	
Irregular contour			
No	9 (20.45)	90 (72.00%)	0.000
Yes	35 (79.45)	35 (28.00%)	
Black striation periprostatic fat			
No	24 (54.55)	111 (88.80)	0.000
Yes	20 (45.45)	14 (11.20)	
Measurable ECE on MRI			
No	24 (54.55)	123 (98.40)	0.000
Yes	20 (45.45)	2 (1.60)	
Retoprostatic angle obliteration			
No	35 (79.55)	125 (100.00)	—
Yes	9 (20.45)	0 (0.00)	
Proposed variables on MRI			
Index lesion length (mm)	17.6 ± 6.0 (7.0; 30.0)	12.8 ± 4.9 (5.0; 32.0)	0.000
Tumor capsular contact length (mm)	19.9 ± 8.8 (0.0; 40.0)	9.8 ± 6.5 (0.0; 26.0)	0.000

Abbreviations: *SE* Standard Error, *OR* Odds Ratio, *CI* Confidence Interval

* PSAD=PSA/Prostate volume

The mean age at the time of surgery was 60.7 and 61.9 years, the mean prostate volume was 38.8 and 42.8 g, and the mean PSA level was 8.0 and 7.0 ng/ml for pECE+ patients and pECE− patients, respectively. The majority of the lesions were classified as PI-RADS 4 or PI-RADS 5, and only 10 lesions were non-aggressive lesions, i.e., PI-RADS 3 lesions.

From the descriptive analysis, we conclude that PSA, PI-RADS v2, prostate volume, PSA density (PSAD), and age did not differ between the two groups. In Table 1, it is apparent that the pECE+ patients had significantly large ILL and TCCL values. Regarding semantic features on MRI, the presence of smooth capsular bulging, capsular disruption, unsharp margin, and irregular contour were analogous between the two groups.

Obliteration of the rectoprostatic angle was not observed in pECE− patients (Table 1). Forty-five percent of pECE+ patients showed measurable ECE on MRI, whereas almost 100 % (98%) of the pECE− patients did not present this feature on MRI. The presence of these two features on MRI are approximately 100% associated with pECE (Table 2).

**Table 2:** Results from univariable and multivariable binary logistic regression models to describe pathological ECE risk

Variable	Univariable Linear Regression Model				Multivariable Linear Regression Model			
	Coef.	SE	OR (95% CI)	*p*-value	Coef.	SE	OR (95% CI)	*p*-value
Intercept	–	–	–	–	−2.701	0.773	0.067 (0.015, 0.305)	0.000
Age at MRI	−0.026	0.025	0.975 (0.927, 1.025)	0.317				
Prostate volume	−0.011	0.010	0.989 (0.970, 1.009)	0.269	−0.033	0.015	0.968 (0.940, 0.996)	0.027
PSA	0.053	0.040	1.055 (0.975, 1.141)	0.186	–	–	–	–
PSAD*	2.210	1.218	9.117 (0.838, 99.178)	0.070	–	–	–	–
Index lesion lenght (ILL)	0.152	0.034	1.164 (1.089, 1.244)	0.000	–	–	–	–
Tumor capsular contact length (TCCL)	0.179	0.032	1.196 (1.124, 1.273)	0.000	0.093	0.047	1.097 (1.001, 1.203)	0.048
Index lesion PI-RADSv2								
More Aggressive (≥4)	1.205	1.069	3.336 (0.410, 27.115)	0.260	–	–	–	–
Smooth capsular bulging								
Yes	2.006	0.508	7.434 (2.749, 20.106)	0.000	–	–	–	–
Capsular disruption								
Yes	2.456	0.478	11.659 (4.573, 29.727)	0.000	1.112	0.576	3.040 (0.983, 9.402)	0.054
Unsharp margin								
Yes	1.810	0.431	6.113 (2.628, 14.220)	0.000	–	–	–	–
Irregular contour								
Yes	2.303	0.424	10.000 (4.360, 22.935)	0.000	–	–	–	–
Black strition periprostatic fat								
Yes	1.888	0.415	6.607 (4.360, 22.935)	0.000	–	–	–	–
Measurable ECE								
Yes	3.937	0.774	51.250 (11.235, 233.774)	0.000	1.652	0.991	5.217 (0.749, 36.357)	0.095
Gleason score								
More aggressive [7 (4 + 3), 8 (4 + 4), 9 (4 + 5), 9 (5 + 4)]	2.481	0.408	11.955 (5.369, 26.620)	0.000	1.823	0.518	6.188 (2.242, 17.081)	0.000

Abbreviations: *SE* Standard Error, *OR* Odds Ratio, *CI* Confidence Interval. * PSAD=PSA/Prostate volume

The association between the covariates under study and the outcome variable was determined using a univariable logistic regression model (Table 2). We observed that GS, TCCL, ILL, and MRI variables were individually associated with the presence of pECE in the surgical specimen with different degrees of relevance (Table 2). The other variables, PSA, PI-RADS v2, prostate volume, PSAD, and age, were not associated with pECE+.

For the multivariable regression model, we selected variables with *p*-values of < 0.15 in a univariable analysis and variables that were clinically significant. Unsharp margin, ILL, and obliteration of the rectoprostatic angle were excluded from the multivariable analysis because they demonstrated high collinearity with capsular disruption, TCCL, and measurable ECE, respectively. Based on the multivariable logistic regression model (Table 2), we found the following to be significant predictors for pECE+: TCCL, capsular disruption, measurable ECE on MRI, and aggressive prostate biopsy GS (Table 2). Notably, prostate volume became a significant variable after adjusting for the other variables under consideration.

For every increase of one millimeter in the capsular contact length, the odds of presenting pECE+ increases by 9% compared with patients with no contact capsular length.

The odds of presenting pECE+ were 3.3, 5.5, and 6.2 times higher in patients with capsular disruption, measurable ECE on MRI, and a GSs of ≥(4 + 3), respectively, compared to patients without these characteristics.

To evaluate the performance of the multivariable logistic regression model, we computed the ROC curve and the AUC. The estimated model shows a good performance in distinguishing pECE+ patients from pECE− patients, with an AUC of 0.90 (86.0–95.8%), a high sensitivity (93%), and moderate specificity (70%) (Table 3).

**Table 3:** Metrics for assessing the quality of the estimated model in test and validation samples

Measure	Value (95% CI)
Test Sample	
Area Under the Curve (AUC)*	90.8 (85.3, 94.5)
Sensitivity	86.4 (80.4, 90.7)
Specificity	78.4 (71.6, 83.9)
Hosmer and Lemeshow test**	4.964 (0.761)
Validation Sample	
Area Under the Curve (AUC)*	85.0 (74.0, 95.8)
Sensitivity	84.2 (72.9, 91.4)
Specificity	82.5 (70.9, 90.1)

Data are given in percentages, with 95% confidence intervals in parenthesis

* Confidence interval was obtained from 2000 stratified bootstrap replicates

** Test statistic; and *p*-value in parenthesis

To test the reproducibility of the model, we employed a validation set of 59 freshly recruited participants. Table 4 presents the clinical and MRI characteristics of both groups, and no differences were observed between the original test cohort and the validation cohort, which proves the stability of the model. Furthermore, ROC curves were calculated for patients in the validation cohort. The AUC for the validation group was 85%, sensitivity was 84%, and specificity was 83%, which are close to the values obtained for the original test group (Table 3).

**Table 4:** Comparing the sample characteristics of the test sample with the validation sample

Variables	Test Sample (n.° of patients = 169)	Validation Sample (n.° of patients = 59)	*p-value*
Continuous variables			
Age at MRI (years)	61.5 ± 6.8 (41.2; 75.8)	62.3 ± 6.4 (43.0; 77.0)	0.472
Prostate volume (g)	41.7 ± 20.1 (18; 148)	47.7 ± 23.1 (19; 150)	0.080
PSA (ng/ml)	7.3 ± 4.1 (2.2; 31.0)	6.9 ± 5.1 (2.0; 38.0)	0.566
PSAD* (ng/ml/g)	0.2 ± 0.1 (0.0; 1.0)	0.2 ± 0.1 (0.0; 0.7)	0.018
Index lesion size (mm)	14.1 ± 5.6 (5.0; 32.0)	13.8 ± 6.7 (6.0; 39.0)	0.796
Capsular contact length (mm)	12.4 ± 8.4 (0.0; 40.0)	12.0 ± 9.6 (0.0; 57.0)	0.767
Categorical variables			
Index lesion PI-RADS V2			
3	10 (5.92)	3 (5.09)	
4	91 (53.84)	37 (62.71)	0.507
5	68 (40.24)	19 (32.20)	
Smooth capsular bulging			
No	66 (39.05)	34 (57.63)	0.015
Yes	103 (60.95)	25 (42.37)	
Capsular disruption			
No	87 (51.48)	37 (62.71)	0.172
Yes	82 (48.52)	22 (37.29)	
Unsharp margin			
No	80 (47.34)	35 (59.32)	0.131
Yes	89 (52.66)	24 (40.68)	
Irregular contour			
No	99 (58.58)	38 (64.41%)	0.445
Yes	70 (41.42)	21 (35.59%)	
Black strition periprostatic fat			
No	135 (79.88)	48 (81.36)	0.956
Yes	34 (20.12)	11 (18.64)	
Measurable ECE			
No	147 (86.98)	51 (86.44)	1.000
Yes	22 (13.02)	8 (13.56)	
Retoprostatic angle obliteration			
No	160 (94.67)	53 (89.83)	0.224
Yes	9 (5.33)	6 (10.17)	
Gleason score			
Less Aggressive [6 (3 + 3), 7 (3 + 4)]	120 (71.01)	36 (61.02)	0.193
More Aggressive [7 (4 + 3), 8 (4 + 4), 9 (4 + 5), 9 (5 + 4)]	49 (28.99)	23 (38.98)	

Each continuous variable is represented as average ± standard deviation (minimum; maximum). Each categorical variable is described by the number of patients in each level (percentage). The *p-values* were obtained by the following tests: two-tailed Fisher’s exact tests for categorical variables; two-independent samples *z*-test (two-tailed tests)

**Fig. 3:**
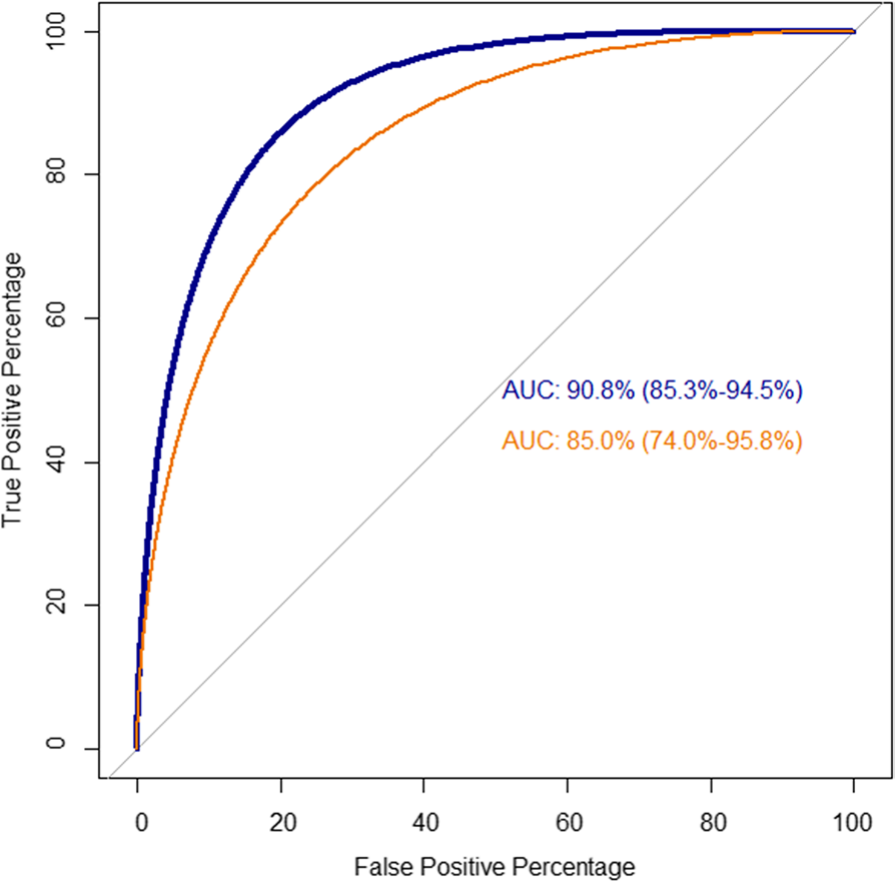
ROC curves for test sample and validation group based on the estimated multivariable logistic model. The ROC curves and AUCs with confidence intervals were obtained from 2000 bootstrap replicates obtained from the test sample (blue line; 169 patients) and validation sample (orange line; 59 patients)

Therefore, the estimated model has excellent performance regarding its ability to distinguish between patients with pECE and patients without pECE (Fig. 3).

**Fig. 4:**
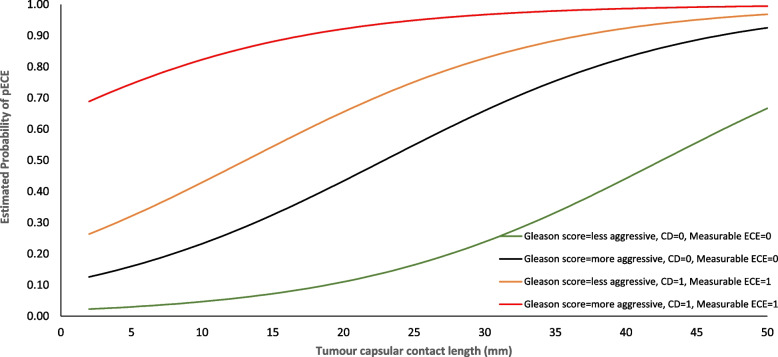
Plot of estimated probability of pECE based on estimated model from Table 2 vs TCCL. TCCL: Tumor Capsular Contact Length. The prostate volume was considered equal to its median value in the test sample (38 g). The lines displayed in the graph show the estimated probabilities according to the values of the binary categorical covariates. The green and black lines show the changes in the probability of pECE+ when the Gleason score varies from less aggressive to more aggressive, keeping absent the remaining categorical variables. The orange and red lines represent the changes in the probability of pECE+ when the Gleason score varies from less aggressive to more aggressive when the remaining categorical variables are present. CD: capsular disruption (0: not present/1: present). Gleason score more aggressive: ≥7(4 + 3); Gleason score less aggressive: <7(3 + 4); Measurable ECE on MRI (0: not present/1: present)

With the estimated model, we calculated the probability of being pECE+ for each patient based on the value of the variables that are part of the model itself. Figure 4 presents the estimated probability of a patient being pECE+ based on the estimated model presented in Table 2 versus the TCCL for a prostate volume of 38 g (which is the median value for the test sample). As expected, measurable ECE on MRI is the strongest predictor of pECE, independent of the other variables, which corresponds with what is already known and the current standard of care (red and orange lines in Fig. 4). However, when this conventional variable was absent, we observed that TCCL and GS were useful for predicting pECE accurately. Thus, based on this model, the estimated probability of being pECE+ for one patient without ECE on MRI or capsular disruption, and a TCCL of 15 mm, is approximately 10% for patients with marginally aggressive GS, and this probability increases to 30% in patients with fully aggressive GS. The difference in the probability of pECE for both groups, i.e., fully aggressive GS (black line) vs. slightly aggressive GS (green line) becomes larger as capsular contact increases (Fig. 4).

To assess the reproducibility of the semantic features, we calculated the agreement between the MRI readings of the two radiologists using the ICC. The variables were considered in agreement between the MRI readers if ICC > 0.75. In our series, the following were the reproducible imaging features: measurable ECE on MRI, TCCL, and ILL. The other MRI features were irreproducible across the two MRI readers with an ICC of < 0.75 (Table 5). Of all the significant variables considered in our model, capsular disruption was the only variable that was not reproducible between the two MRI readers. When we excluded capsular disruption from the final model, the AUC remained approximately the same (0.9). The estimated model has good performance and is also reproducible.

**Table 5:** Inter-reader agreement for MRI semantic features

Sematic feature	ICC *	95% Confidence Interval
Black striation periprostatic fat	0.71	(0.60, 0.79)
Retoprostatic angle obliteration	0.68	(0.75, 0.87)
Measurable ECE on MRI	0.88	(0.55, 0.76)
Smooth capsular bulging	0.28	(0.02, 0.48)
Capsular disruption	0.59	(0.44, 0.70)
Unsharp margin	0.47	(0.28, 0.61)
Irregular contour	0.61	(0.47, 0.72)
TCCL (tumor capsular contact length)	0.82	(0.75, 0.86)
ILL (index lesion length)	0.82	(0.75, 0.87)
PI-RADS v2 score	0.73	(0.47, 0.66)

*ICC: Intraclass Correlation

## Discussion

In this study, we develop a model for predicting pECE+ based on only four characteristics: prostate biopsy GS, two classical semantic features on MRI (measurable ECE on MRI and capsular disruption), and TCCL. This model is accurate and reproducible, with goodness-of-fit proved by its AUC, which is 85% for the validation group and approximately 90% for the test group.

First, we evaluated the impact of each classical semantic MRI feature proposed by the ESUR for predicting pECE+, which can be categorized into *early signs of ECE*: capsular irregularity, capsular bulging, and unsharp margin, and *late signs of ECE*: obliteration of the rectoprostatic angle, invasion of periprostatic fat, and measurable ECE on MRI. These *late signs of ECE* are very uncommon in pECE− patients, as we have proven and as reported by other similar studies [12, 13, 18]. These *late signs of ECE* indicate the presence of significantly invasive clusters of neoplastic cells, which produce irregular disruption of the prostate capsule, and subsequently, infiltration into periprostatic fat, which is observed in advanced PCa stages [12]. In our study, almost all patients with measurable ECE were pECE+, excluding only two patients (1.6%). On reviewing these two false-positive cases, we concluded that in one case, there was a hemorrhage hampering the interpretation of the images, and in the other, there was granulomatous prostatitis coexisting with the PCa. When measurable ECE is identified on MRI, the radiologist can report the presence of pECE with a high degree of confidence. However, measurable ECE is a relatively late marker of pECE and becomes visible predominantly in the advanced stages of PCa. Therefore, it should be reckoned that its absence does not rule out pECE. At our institution, the majority of pECE+ cases operated on had minimal (< 5 mm) pathologic periprostatic extension. This explains why measurable ECE is observed in only 45% of all pECE+ cases.

The critical feature in our model is the introduction of TCCL and a prostate biopsy GS of ≥7 (4 + 3) as significant predictors of pECE+ in unadvanced cases. We proved that TCCL is an independent and reproducible predictor of pECE+, which corroborates a recent review conducted by Kim et al. [19]. The optimal cutoff value for predicting pECE has not yet been established by previous studies and varies between 10 mm and 20 mm [19]. We posit that the global assessment of TCCL and its integration into the model and the other covariables of the model is the key for obtaining the individual probability of being pECE+. Figure 4 presents a demonstration of the probability of being pECE+ calculated using our predictive model. Measurable ECE on MRI is the strongest predictor of pECE, with an approximately 80% probability of the patient being pECE+ when present alone. TCCL and prostate biopsy GS take on a significantly relevant role when measurable ECE and capsular disruption are absent (Fig. 4: black and green lines). For example, in patients with an aggressive prostate biopsy GS, with no measurable ECE or capsular disruption observed on MRI, and a TCCL of > 25 mm, the probability of being pECE+ is greater than 50%. With the same imaging features, but with a marginally aggressive prostate biopsy GS, the TCCL cutoff would need to be increased to approximately 40 mm to achieve a similar probability of being pECE+, i.e., > 50%. Only [18] developed a predictive grading system for predicting pEPE called MRI-derived EPE (MRI-EPE) grade, which combines MRI semantic imaging features and TCCL into three grades. The MRI-EPE grade 1 corresponds to TCCL > 1.5 cm, with a 24% risk of being pEPE+. This score does not account for the influence of the GS and MRI images on the final prediction, which makes it dissimilar from our estimation model. We prove that the same TCCL value (> 1.5 cm) corresponds to different risk levels of being pEPE+ depending on the aggressivity of the GS. For example, in our estimation model, the estimated probability of being pECE+ for a patient with a TCCL of 15 mm is approximately 10% with a marginally aggressive GS, and the probability increases to 30% for patients with a fully aggressive GS. In contrast, [18] did not assess the inter-reader variability of their MRI grading system.

With these predictors (GS and TCCL), our model can diagnose more patients with pECE+ who have no measurable ECE on MRI in the early stages of PCa, improving the global sensitivity to 86% and maintaining moderate-to-high specificity at approximately 73%. These values are in agreement with a recent meta-analysis by Kim et al., which reports a sensitivity and specificity of 0.79 and 0.67, respectively, and an AUC of 0.81 [19]. However, this differs slightly from the meta-analysis by De Rooij et al., which reports a sensitivity and specificity of 0.57 and 0.91, respectively, meaning that the MRI scan had a high specificity but poor and heterogeneous sensitivity for local PCa staging [10]. The differences in these results are attributable to the use of objective parameters such as TCCL in combination with subjective semantic features to predict pECE+ in the studies analyzed by Kim et al. contrasted with the studies included in the meta-analysis by De Rooij et al., which used only subjective semantic features to predict pECE+. The features in the former may have crucial clinical implications, as a high sensitivity or highly specific interpretation may be preferred, depending on the clinical scenario. For example, high sensitivity is required when selecting patients for enrollment in active surveillance programs or choosing candidates for RP with neurovascular bundle sparing. On the other hand, high specificity could be favored when the objective is to avoid potential curative treatment delays. This model shows a good performance to select patients in the early stages of the disease who are the best candidates for RP with neurovascular bundle sparing reducing the side effects conditioned by more invasive surgery. Accurate pre-operative of pECE can also change the therapy decision leading to the recommendation of adjuvant radiotherapy after RP.

Consistent with the literature, our model confirms that the combination of MRI features with a pre-treatment biopsy GS is superior to imaging features alone for predicting pECE [20]. The GS improves the diagnostic accuracy regarding the aggressivity of the disease, and it has been incorporated into many predictive nomograms for detecting ECE [14, 21].

The clinical covariates of age and index lesion PI-RADS score were not associated with pECE (*p*-values > 0.10), as reported in previous studies [18].

The covariate, PSA levels, was also not significant, even when categorized in the following groups: < 10, 10–20, and > 20. In our study, PSA was not selected for predicting pECE, which is dissimilar from the specifications of existing classical cancer nomograms extensively used in the literature, such as Cancer of the Prostate Risk Assessment (CAPRA), Partin tables, and the Memorial Sloan Kettering Cancer Center (MSKCC) nomogram [21–23]. PSA is also used in more recent nomograms based on MRI and Briganti nomograms as a preoperative parameter influencing ECE prediction [24]. The results of our study might be related to the small number of patients with high PSA levels in our sample data: only four patients had PSA levels over 20 ng/ml, which is insufficient for detecting statistical differences between pECE+ patients and pECE− patients.

Our model presents good agreement between the MRI readers for the presence of TCCL and measurable ECE (ICC of 0.82 and 0.88, respectively). However, there was insufficient agreement regarding capsular disruption. The other imaging covariables are extremely heterogeneous between readers and were not significant in the multivariable model.

Our study has some limitations. First, the study sample is relatively small, and the study was performed at a single center using the same MRI protocol and a 3 T MRI scanner. To overcome this bias, we introduced a validation sample of 59 patients whose MRI examinations were performed outside the primary study facility and with different MRI equipment to increase the robustness of the model. We did not identify statistical differences between the test and validation groups (Table 4) for the variables under consideration. Nevertheless, this result does not allow to conclude that the heterogeneity between MRI’s acquisitions protocol and technical specifications of the equipment in the validation sample could modify interpretation accuracy. Furthermore, there is a sampling bias resulting from the selection of prostatectomy specimens as the histopathological reference standard because prostatectomy was not proposed for the significantly advanced cases.

Second, we focused only on the index lesion identified on MRI, and it was the only one correlated with the prostate lesion. We did not take tumor multifocality on MRI and pathology into account.

Third, the TCCL measurements presented in our study may be limited by our institutional software and sequence specifications and require validation at other institutions and the use of other MRI protocols.

Our model uses measurable ECE as a determinant MRI semantic feature to detect pECE. Although measurable ECE is correlated between readers, it has a low prevalence in pECE+ patients because it is observed predominantly in significantly advanced cases. The other considerably prevalent semantic feature (capsular disruption) is not significant for detecting early-stage cases of pECE and is not so correlated between readers. Hence, we need to conduct additional research on preoperatively detecting microscopic ECE using a grading of objective markers, such as TCCL and GS, and validate these markers at various institutions or incorporate a new artificial intelligence (AI) analysis into the estimation model developed in this study. A computer-based method of extracting and analyzing image features qualitatively (Radiomics) could provide more information about the PCa tumor facilitating risk stratification and therapeutic management of these patients. MRI has been the most studied imaging modality for radiomics application in PCa, so far, but more research is warranted in order to get robustness of MRI- based radiomics features models [25].

## Conclusion

We put forward a robust MRI model based on correlated MRI features (measurable ECE) combined with prostate biopsy GS and TCCL. The proposed model has good accuracy and high sensitivity for detecting pECE, making it a valuable tool for urological surgeons in designing the ideal nerve-sparing prostate cancer surgery.

## Data Availability

The datasets used and/or analysed during the current study are available from the corresponding author on reasonable request.

## References

[1] Global Cancer Observatory [Internet]. [cited 2020 Feb 16]. Available from: https://gco.iarc.fr/.

[2] Pina F, Castro C, Ferro A, Bento MJ, Lunet N (2017). Prostate cancer incidence and mortality in Portugal: trends, projections and regional differences. Eur J Cancer Prev.

[3] Talab SS, Preston MA, Elmi A, Tabatabaei S (2012). Prostate Cancer imaging: what the urologist wants to know. Radiol Clin N Am.

[4] Hubanks JM, Boorjian SA, Frank I, Gettman MT, Thompson RH, Rangel LJ (2014). The presence of extracapsular extension is associated with an increased risk of death from prostate cancer after radical prostatectomy for patients with seminal vesicle invasion and negative lymph nodes. Urol Oncol Semin Orig Investig.

[5] Johnson LM, Turkbey B, Figg WD, Choyke PL (2014). Multiparametric MRI in prostate cancer management. Nat Rev Clin Oncol.

[6] Weinreb JC, Barentsz JO, Choyke PL, Cornud F, Haider MA, Macura KJ, et al. PI-RADS Prostate Imaging – Reporting and Data System: 2015, Version 2. Eur Urol [Internet]. 2016 Jan;69(1):16–40. Available from: https://linkinghub.elsevier.com/retrieve/pii/S0302283815008489. 10.1016/j.eururo.2015.08.052 PMC6467207 26427566

[7] Barentsz JO, Weinreb JC, Verma S, Thoeny HC, Tempany CM, Shtern F, et al. Synopsis of the PI-RADS v2 Guidelines for Multiparametric Prostate Magnetic Resonance Imaging and Recommendations for Use [Internet]. Vol. 69, European Urology. 2016. p. 41–9. Available from: https://linkinghub.elsevier.com/retrieve/pii/S0302283815007836. 10.1016/j.eururo.2015.08.038 PMC6364687 26361169

[8] Engelbrecht MR, Jager GJ, Laheij RJ, Verbeek ALM, van Lier HJ, Barentsz JO. Local staging of prostate cancer using magnetic resonance imaging: a meta-analysis. Eur Radiol. 2002;12(9):2294–302. Available from: http://link.springer.com/10.1007/s00330-002-1389-z. 10.1007/s00330-002-1389-z 12195484

[9] da Silva RC, Sasse AD, Matheus WE, Ferreira U (2013). Magnetic resonance image in the diagnosis and evaluation of extra-prostatic extension and involvement of seminal vesicles of prostate cancer: a systematic review of literature and meta-analysis. Int Braz J Urol.

[10] de Rooij M, Hamoen EHJ, Witjes JA, Barentsz JO, Rovers MM (2016). Accuracy of magnetic resonance imaging for local staging of prostate cancer: a diagnostic meta-analysis. Eur Urol.

[11] Barentsz JO, Richenberg J, Clements R, Choyke P, Verma S, Villeirs G (2012). ESUR prostate MR guidelines 2012. Eur Radiol.

[12] Pesapane F, Standaert C, De Visschere P, Villeirs G (2020). T-staging of prostate cancer: identification of useful signs to standardize detection of posterolateral extraprostatic extension on prostate MRI. Clin Imaging.

[13] Schieda N, Quon JS, Lim C, El-Khodary M, Shabana W, Singh V (2015). Evaluation of the European Society of Urogenital Radiology (ESUR) PI-RADS scoring system for assessment of extra-prostatic extension in prostatic carcinoma. Eur J Radiol.

[14] Shieh AC, Guler E, Ojili V, Paspulati RM, Elliott R, Ramaiya NH (2020). Extraprostatic extension in prostate cancer: primer for radiologists. Abdom Radiol.

[15] Robin X, Turck N, Hainard A, Tiberti N, Lisacek F, Sanchez J-C, Muller M (2011). pROC: An open-source package for R and S+ to analyze and compare ROC curves. BMC Bioinformatics..

[16] Youden WJ (1950). Index for rating diagnostic tests. Cancer..

[17] Hosmer DW, Lemeshow S, Sturdivant RX (2013). Applied logistic regression. Wiley series in probability and statistics.

[18] Mehralivand S, Shih JH, Harmon S, Smith C, Bloom J, Czarniecki M (2019). A grading system for the assessment of risk of extraprostatic extension of prostate cancer at multiparametric MRI. Radiology..

[19] Kim TH, Woo S, Han S, Suh CH, Ghafoor S, Hricak H (2020). The diagnostic performance of the length of tumor capsular contact on mri for detecting prostate cancer extraprostatic extension: a systematic review and meta-analysis. Korean J Radiol..

[20] Patel KM, Gnanapragasam VJ (2016). Novel concepts for risk stratification in prostate cancer. J Clin Urol.

[21] Eifler JB, Feng Z, Lin BM, Partin MT, Humphreys EB, Han M (2013). An updated prostate cancer staging nomogram (Partin tables) based on cases from 2006 to 2011. BJU Int.

[22] Brajtbord JS, Leapman MS, Cooperberg MR (2017). The CAPRA score at 10 years: contemporary perspectives and analysis of supporting studies. Eur Urol.

[23] Milonas D, Venclovas Z, Muilwijk T, Jievaltas M, Joniau S. External validation of memorial Sloan Kettering Cancer center nomogram and prediction of optimal candidate for lymph node dissection in clinically localized prostate cancer. Cent Eur J Urol. 2020;73(1):19–25. Available from: http://ceju.online/journal/2020/prostate-cancer-lymph-node-invasion-preoperative-MSKCC-nomogram-2021.php. 10.5173/ceju.2020.0079 PMC7203765 32395318

[24] Gandaglia G, Ploussard G, Valerio M, Mattei A, Fiori C, Fossati N (2019). A novel nomogram to identify candidates for extended pelvic lymph node dissection among patients with clinically localized prostate Cancer diagnosed with magnetic resonance imaging-targeted and systematic biopsies. Eur Urol.

[25] Spohn SKB, Bettermann AS, Bamberg F, Benndorf M, Mix M, Nicolay NH (2021). Radiomics in prostate cancer imaging for a personalized treatment approach - current aspects of methodology and a systematic review on validated studies. Theranostics.

