# Correction: Early biomarkers of extracapsular extension of prostate cancer using MRI-derived semantic features

**DOI:** 10.1186/s40644-023-00550-1

**Published:** 2023-04-03

**Authors:** Adalgisa Guerra, Filipe Caseiro Alves, Kris Maes, Steven Joniau, João Cassis, Rui Maio, Marília Cravo, Helena Mouriño

**Affiliations:** 1 Radiology Department, Hospital da Luz Lisboa, Avenida Lusíada, N° 100, 1500-650 Lisbon, Portugal; 2 Faculty of Medicine, Clinical Research CIBIT/ ICNAS, University of Coimbra, 3000-548 Coimbra, Portugal; 3 Urology Department, Hospital da Luz Lisboa, Avenida Lusíada, N° 100, 1500-650 Lisbon, Portugal; 4 Urology Department, University Hospitals Leuven, UZ Leuven Gasthuisberg Campus, Urology, Herestraat 49, 3000 Leuven, Belgium; 5 Pathology Department, Hospital da Luz Lisboa, Avenida Lusíada, N° 100, 1500-650 Lisbon, Portugal; 6 Nova Medical School-Nova University of Lisbon, Portugal e Hospital da Luz Lisboa, Campo Mártires da Pátria, N° 130, 1169-056 Lisbon, Portugal; 7 Gastroenterology Department- Hospital da Luz Lisboa, Avenida Lusíada, N° 100, 1500-650 Lisbon, Portugal; 8 Centro de Estatística E Aplicações, Departamento de Estatística E Investigação Operacional, Faculdade de Ciências, Universidade de Lisboa, Edifício C6 – Piso 4, Campo Grande, 1749 – 016 Lisbon, Portugal

**Correction: Cancer Imaging 22, 74 (2022)**

**https://doi.org/10.1186/s40644-022-00509-8**

The original publication of this article [1] contained several consistency errors. The incorrect and correct information is shown below

**Abstract**

**Incorrect**

- The study participants included 184 patients who had undergone RARP at our institution, 26% of whom were pECE + (i.e., pECE positive). Significant predictors of pECE + were TCCL, capsular disruption, measurable ECE on MRI, and a GS of ≥ 7(4 + 3) on a prostate biopsy.

**Correct**

- The study participants included **185** patients who had undergone RARP at our institution, 26% of whom were pECE + (i.e., pECE positive). Significant predictors of pECE + were TCCL, capsular disruption, measurable ECE on MRI, and a GS of ≥ 7(4 + 3) on a prostate biopsy.

**Materials and methods**

**Incorrect**

- This study included 257 participants, all of whom were patients diagnosed with PCa between 2015 and 2018.
- The exclusion criteria (Fig. 1) led to 72 patients being excluded, leaving only 185 patients for analysis.

**Correct**

- This study included **237** participants, all of whom were patients diagnosed with PCa between 2015 and 2018.
- The exclusion criteria (Fig. 1) led to 52 patients being excluded, leaving only 185 patients for analysis.

**Results**

**Incorrect**

- For every increase of one millimeter in the capsular contact length, the odds of presenting pECE + increases by 9% compared with patients with no contact capsular length.

**Correct**

- For every increase of one millimeter in the capsular contact length, the odds of presenting pECE + increases by **9.7%** compared with patients with no contact capsular length.

**Incorrect**

- The odds of presenting pECE + were 3.3, 5.5, and 6.2 times higher in patients with capsular disruption, measurable ECE on MRI, and a GSs of ≥ (4 + 3), respectively, compared to patients without these characteristics.

**Correct**

- The odds of presenting pECE + were **3.0, 5.2**, and 6.2 times higher in patients with capsular disruption, measurable ECE on MRI, and a GSs of ≥ (4 + 3), respectively, compared to patients without these characteristics.

**Incorrect**

- To evaluate the performance of the multivariable logistic regression model, we computed the ROC curve and the AUC. The estimated model shows a good performance in distinguishing pECE + patients from pECE − patients, with an AUC of 0.90 (86.0–95.8%), a high sensitivity (93%), and moderate specificity (70%) (Table 3).

**Correct**

- To evaluate the performance of the multivariable logistic regression model, we computed the ROC curve and the AUC. The estimated model shows a good performance in distinguishing pECE + patients from pECE − patients, with an AUC of 0.91 (85.3–94.5%), a high sensitivity (86%), and moderate specificity (78%) (Table 3).

**Figure 4 caption**

**Incorrect**

- Gleason score less aggressive: < 7(3 + 4)

**Correct**

- Gleason score less aggressive: < 7 (4 + 3)
